# Medical Opinion and Movement

**Published:** 1908-04-11

**Authors:** 


### Medical Opinion and Movement.

Hydrogen peroxide has been more enthusiastically advocated and employed by surgeons in France as an antiseptic and purifying agent than in any other country. M. Masotti now proclaims its virtue as a means of removing chloasma, freckles, and other pigmentations. He first makes fine scarifications every five days very close together on the spot which it is desired to remove. The part is then washed with warm water, and a tampon of cotton-wool soaked in hydrogen peroxide is bandaged on. Erythema is produced on the following day, and slight desquamation follows a few days later. The pigmentation rapidly disappears. According to M. Masotti the scarification causes renovation of the epidermis, and the oxygen is brought into direct contact with the pigment and exercises a decolourising effect. It is claimed that the treatment is without pain or irritation, and a cure can be effected with a few sittings, but it is admitted that occasionally the pigmentation has recurred, especially when some general condition such as anæmia is present.

---

The specific micro-organism of scarlet fever still baffles the researches of bacteriologists. It has for some time been assumed that the disease was caused by a specific strain of streptococcus, but extensive research has failed to associate any particular strain of this coccus with the disease. It is now generally held that these streptococcal infections of the glands, ears, and other organs are secondary only to the primary infection, which at present remains undiscovered. Quite recently Dr. Harold Kerr has carried out an extensive bacteriological examination of scarlet fever cases, making cultures from the throat, from nasal and aural discharges, from abscesses and from the blood. His results confirm those of Andrews and Horder in regard to the variety and kind of organisms isolated, including many strains of streptococci, but he has failed to associate the disease with the presence of any particular microbe. In spite, however, of this failure to isolate the specific microbe, several attempts have been made to obtain a curative serum for the disease. Pulawski, of Radziejow, has used a serum since 1904, and claims for it considerable therapeutic value. By comparing a series of cases treated with and without the serum he is able to show a reduction of mortality in severe cases from 41.6 to 14.5 per cent. More recently Marpmann has obtained a serum by injecting animals with a product from the scales of desquamation. The serum is termed scarlatin, and is administered by the mouth in 3 to 8 minims in milk. Professor Monti, of Vienna, and several other Continental authorities report good results from the use of this serum.

---

Leprosy is one of those scourges from which man has long yearned to be freed by the discovery of a cure. When Hansen demonstrated the causative germ of the disease it was hoped that this might be followed by the elaboration of some antitoxic or bactericidal serum, but the failure to cultivate the organism outside the human body has so far obstructed research on these lines. In a recent lecture, however, delivered at the London School of Tropical Medicine, Professor Deycke has proclaimed a new specific treatment. This treatment is the result of long researches carried out as Director of the Military School of Practical Medicine at Constantinople. Although unable to cultivate the lepra bacillus, Professor Deycke succeeded in isolating from several severe cases of nodular leprosy a micro-organism which he terms streptothrix leproides. From cultures of this organism he extracted a fatty substance which he terms "nastin," and which, injected into the body in conjunction with benzoyl chloride in due proportions, arrests the disease, and in some cases apparently effects a cure. The nastin and benzoyl chloride appear to kill the lepra bacilli by depriving them of their fatty contents, and so render them an easy prey to the tissue elements of the body. Such is a rough outline of the treatment, but a detailed account of the researches by which these results were obtained is well worthy of perusal, and shows that the treatment is based upon thoroughly scientific experimental work. Professor Deycke makes no excessive claims for the treatment, but he is able to record some very successful results. He is now in communication with the Colonial Office with a view to obtain permission to try the treatment on a large scale in some of the leper asylums in British colonies.

---

The human subject is always the most unreliable factor, the weak point, in the smooth working of any mechanical system, be it for locomotion or other purpose. In spite of the most carefully devised automatic safeguards, human judgment and control can never be entirely eliminated, and it is here where the breakdown is likely to take place. Where, as in the case of locomotion, the lives of thousands are dependent upon the correct judgment of a few, it is of the greatest importance to ensure that those few are properly chosen for the work, and that the functions of the body they are called upon to exercise are ascertained to be normal by properly devised tests. It has recently been clearly demonstrated by several authorities qualified to speak on the subject that the official tests used by the Board of Trade to discover colour-blindness are most unsatisfactory and based on entirely wrong principles, and one is led to the uncomfortable conviction that men who hold certificates for good vision may actually possess serious defects in this respect. A contemporary suggests that many railway accidents may be accounted for by the inability of the engine-driver to distinguish a green from a red light under certain abnormal conditions, and cites the recent accident on the North-Eastern Railway at Guisborough as a possible case in point. It is pointed out that the evidence given by Wheatley, the engine-driver, that the distant signal "showed partly green and partly red," and that he ran past the near home and home signals without seeing them, strongly suggests imperfect vision. A careful examination of the driver's vision might explain the cause of this accident, which, in the words of the report, is "inexplicable" and due to "want of care on Driver Wheatley's part," but no such examination appears to have been made.

In private practice there are few more difficult cases to treat than those of epilepsy, even in a mild form. Someone has said, truthfully enough, that where a variety of drugs are advised in the treatment of any special disorder, one may take it for granted that no remedy is known to have a constant curative influence upon the disorder in question. And the number of remedies vaunted in the treatment of epilepsy is almost legion. Hippocrates, Paulus Ægинitus and the Salernians tabled many drugs, and in modern days the number has been very largely added to. Leubusches has recently attempted to summarise the methods of treatment, and his paper makes interesting reading, although the author does not enter into the history of the therapeutics of epilepsy, a subject which in itself would make an interesting article.

All cases of epilepsy, it is needless to say, should be very carefully examined, particular attention being paid to any possible cause of "reflex epilepsy," such as disease of the ear, nose or throat. Syphilitic or other intoxications, such as lead, alcohol, etc., should be considered as probable causative factors, and eliminated, if possible, before the case is accepted as one of idiopathic origin and treated as such. Of all the vaunted drugs the bromides, in the author's opinion, still remain in the front rank. Their use the profession owes, as it is interesting to remember, to a French quack, whose "patent medicine" was analysed by the staff at the Salpêtrière and imitated as closely as possible by means of a strong solution of sodium bromide. How bromides act is still a mystery. Various explanations have been offered, and it is possible that the bromine to a large extent replaces the chloride in the body tissues. It has, at least, been found that the brain tissue of a patient who has been taking bromine for a long time is comparatively free from chlorides, but the subject demands further investigation. Toulouse and Richet have tried to produce "bromine saturation" as quickly as possible by replacing the salt in the normal diet entirely by bromides. The patient gets a salt free diet, and takes a mixture of sodium and potassium bromide as condiment. Balnit, again, put his patients on bromopan, which is merely a special bread, baked with bromides instead of with chlorides. This strictly salt-free diet has now been generally abandoned in favour of less drastic measures.

Usually moderate doses of bromides are found more effective than enormous doses. It is of little use, however, even in the case of children, to give doses smaller than three grains. Leubusches recommends moderate doses of potassium bromide in preference to the sodium salt, which theoretically contains more bromine, but is clinically much less active. As adjuvants, especially in cases where the potassium compound is badly tolerated, he uses a combination of bromine with sesame oil, the so-called bromipin. The Flechsig-opium method he does not favour, but speaks well of the Bechterew method, in which adonis vernalis infusion is combined with sodium bromide. Digitalis and strophanthus are useful drugs in some cases, while belladonna and atropin are indicated in others. Zinc salts and borax, chloral hydrate, amidoacetic acid, amyl hydrate, and the anti-epileptic serum of Cenis have been serviceable in a few cases, but are not to be regarded as equivalent to a regular course of bromides. Finally the author discusses the prophylactic and hygienic treatment of epilpetics.

Arnsperger, who is Professor Narath's principal assistant at the Heidelberg surgical clinic, discusses the diagnosis and treatment of acute cholecystitis in a recent number of the *Medizinische Klinik*. The main questions which the practitioner has to answer in dealing with a doubtful case of gallbladder inflammation are these: Is it a case of cholecystitis? If so, should the treatment be medicinal or surgical? If surgical, which operation is better, cholecystostomy or cholecystectomy? To point out the difficulty of diagnosing such cases, the author states that 28 per cent. of the cases of acute cholecystitis during the last five years at the Heidelberg surgical clinic were wrongly diagnosed. Appendicitis was usually diagnosed. Jaundice is never present at the commencement of an acute cholecystitis, but it occurs when there is a definite infection of the portal system, and its presence is of very unfavourable prognostic significance. Early operation in cases of appendicitis is not the rule at Heidelberg, and the author is of opinion that cases of acute cholecystitis should be treated very much on the same lines as cases of appendicitis, leaving operation until the severity of the acute attack has passed off. Complete rest in bed, with warm applications to the abdomen, usually suffices to improve the patient's condition. Morphia may be given, but cautiously, as it often, as in the case of abdominal conditions, obscures the case. Purgatives should on no account be given, but in cases where the lower bowel is overloaded with fæces oil enemata may be of use. Only water is given by mouth, nutrient enemata or salines being administered. Under such treatment the attack is usually successfully combated.

The author, however, admits that in certain conditions, prompt operation is imperative. The absolute indication is when the attack is so acute, or so progressive that the patient's life is directly endangered by it; the relative indication when palliative treatment has not succeeded in overcoming the acute symptoms. The former indication is made evident when signs of peritonitis or of septic cholangitis manifest themselves, as shown by a rising pulse and an increased tenderness of the abdominal wall, by rigors and by jaundice. As to choice of operation, the author pleads strongly for cholecystectomy. During the past three years thirty-six cases were treated at the Heidelberg clinic, with eight deaths. Four cases were treated by open incision, with one death; twelve by cholecystostomy with three deaths; twelve by cholecystectomy, with one death; and six by cholecystectomy and liver drainage, with three deaths. The death-rate for all operations was thus 22 per cent., a figure which we think compares very favourably with that of London hospitals. Similar results have been obtained by Goldammer and Courvoisier, and there can be little excuse for the statement so frequently made that cholecystostomy is a safer operation than extirpation of the gall-bladder. English authors rely, apparently, on the old statistics of Dr. Mayo. It would be well to collect all available figures and to settle the question, so far as statistics can do so. Cholecystectomy seems to be the only reasonable operation in such cases. No one would think of doing an appendicostomy in cases of appendicitis in preference to an appendicectomy, but all our text-books continue to give the preference to cholecystostomy as "the easier and safer operation."

---

Creighton Wellman, of Angola, West Africa, contributes to the *Journal of Tropical Medicine and Hygiene* an interesting paper on the ætiology of goitre. After reviewing the various current theories regarding the causative factors in this affection he subjects them to criticism in the light of experience gained by practice among the West African natives. Parenchymatous goitre, he concludes, "is probably a paraphenomenon, or possibly a sequel of some pathological process showing no further well-defined symptoms or coarse anatomical changes, and which is dependent on some germ or other cause which in turn depends on particular conditions only perfectly fulfilled in certain localities. . . . The idea of goitre as a secondary manifestation is not far-fetched. In diphtheria, paralysis comes on after the acute symptoms have subsided. In plague, the adenitis is subsequent to gastro-intestinal lesions. In beri-beri we have a more complete analogy, as in this disease the profound and destructive nervous sequelæ are dependent on a slight initial duodenitis which in itself would not attract any attention. It is quite possible that goitre, like beri-beri, is due to an organism which, multiplying in some other part of the body, slowly secretes an extra cellular toxin which the thyroid gland tries to remove from the blood, and in the attempt is subjected to the irritation and chronic inflammation leading to the hyperplasia known as goitre." The paper, though short, will repay perusal, and is an informative contribution to the literature on the subject. The ætiology of this condition is so little understood that any new light upon it deserves careful attention.

---

Marcel Mangin has devoted himself to a clinical study of the Lourdes cures, and has contributed a most readable article to the *Annals of Psychical Science*. The cases cited as cures are not to be included in the category of so-called "functional diseases." In these the cures by faith-healing are perfectly well known and admitted, but medical science has not yet admitted the possibility of definite organic lesions, such as an ununited fracture, being cured by suggestion. Nor are the instances cited by the author absolutely unequivocal. It is true he has given us an account of the autopsy and the clinical history of Derudder, who was "cured" of an ununited fracture of the tibia, but the story of the cure is related by lay witnesses, and the detailed account of the examination at the Bureau des Constatations Medicales is wanting. It would be interesting indeed to have a *résumé* of the reports of this office, edited by an impartial outsider who is in a position to verify the statements and to follow up the patients; but as it stands M. Mangin's account, interesting as it is, does not carry conviction. The cases of Yvonne Aumaitre and Gargam are particularly interesting and particularly annoying to the medical reader, because they are detailed with so little really helpful information. In a discussion of such cases it is highly desirable that extraneous testimony—that is, the evidence of prejudiced lay witnesses—should enter as little as possible, and that the clinical history and anamnesis of the patients should be given with more than ordinary accuracy. This has not been done in the present instance, and M. Mangin's paper, therefore, does not carry us very far. Every medical man is prepared to admit the possibility of "suggestion cures" in certain cases where no real organic lesion is demonstrable, but before we are prepared to accept as cures cases in which it is alleged that the patients had a definite pathological defect, such as a cancer, a tuberculous ulcer, an osteomyelitis, or an ununited fracture, it is necessary that the clinical evidence of the existence of such a lesion should be conclusive. Hitherto, we believe, the Bureau des Constatations has not published any unequivocal cases, and those which are now summarised in M. Mangin's paper are by no means such as a medical society would accept on the evidence given.

---

Caro has summarised the results obtained in Israel's clinic at Berlin in various anæsthetic cases. In this private hospital various methods have been tried to induce anæsthesia, and several different methods are at present in use. Chloroform still maintains its position in the front rank. It is given by the open method, either on a Schimmelbusch's mask or on a towel according to the Edinburgh fashion, and has so far been very popular. The mixture of chloroform vapour and oxygen, given with a Michaeli mask, has not impressed the author, who regards it as dangerous and liable to produce unpleasant after-effects, besides being uncertain and expensive. Scopolamine-morphine injections were tried in 134 cases, but the author does not recommend this method, which, in his opinion, is liable to cause serious intestinal atony. The American (Abbott) method in which the scopolamine is replaced by the less dangerous cactin, was not tried. Lumbar anæsthesia is rapidly winning favour, and was employed in fifty-four cases. In six cases it was a failure, chiefly owing to the fact that the patients were agitated and highly strung. Stovaine and novocaine were used, but at present tropacocaine has entirely replaced both. Lumbar anæsthesia, the author concludes, is specially suitable for women and old persons. In one case only was hypnotism employed to induce anæsthesia.

